# Realising the right to sexual and reproductive health: access to essential medicines for medical abortion as a core obligation

**DOI:** 10.1186/s12914-018-0140-z

**Published:** 2018-02-01

**Authors:** Katrina Perehudoff, Lucía Berro Pizzarossa, Jelle Stekelenburg

**Affiliations:** 1 University Medical Center Groningen, Department of Health Sciences - Global Health Unit, University of Groningen, Groningen, The Netherlands; 2 Global Health Law Groningen Research Centre, Transboundary Legal Studies Department, University of Groningen, Groningen, The Netherlands; 3 MYSU (Women & Health), Montevideo, Uruguay; 4 Medical Centre Leeuwarden, Department Obstetrics and Gynecology, Leeuwarden, The Netherlands; 5 Comparative Program on Health & Society, Munk School of Global Affairs, University of Toronto, Toronto, Canada

**Keywords:** Abortion, Sexual and reproductive health, Right to health, Essential medicines, Access to medicines

## Abstract

**Background:**

WHO has a pivotal role to play as the leading international agency promoting good practices in health and human rights. In 2005, mifepristone and misoprostol were added to WHO’s Model List of Essential Medicines for combined use to terminate unwanted pregnancies. However, these drugs were considered ‘complementary’ and qualified for use when in line with national legislation and where ‘culturally acceptable’.

**Discussion:**

This article argues that these qualifications, while perhaps appropriate at the time, must now be removed. First, compelling medical evidence justifies their reclassification as a ‘core’ essential medicine. Second, continuing to subjugate essential medicines for medical abortion to domestic law and cultural practices is incoherent with today’s human rights standards in which universal access to these medicines is an inextricable part of the right to sexual and reproductive health, which should be supported and realised through domestic legislation.

**Conclusion:**

This article shows that removing such limitations will align WHO’s Model List of Essential Medicines with the mounting scientific evidence, human rights standards, and its own more recently developed policy guidance. This measure will send a strong normative message to governments that these medicines should be readily available in a functioning and human-rights-abiding health system.

## Background

Complications of unsafe abortion are still among the top five causes of direct maternal mortality. Maternal mortality claims the lives of 289,000 women annually, while complications during childbirth result in 5.8 million serious injuries every year and deaths due to unsafe abortion remain close to 13% of all maternal deaths [1]. Unsafe abortion remains a serious human rights and public health problem which is estimated to cause 47,000 maternal deaths and five million maternal disabilities annually [1]. Improving access to family planning and to safe abortion services prevents injury and death that affects women trying to terminate unwanted pregnancies [2–4].

WHO has a primary role to play as the leading international agency promoting good practices in health and human rights. The year 2005 marked a major milestone for access to safe abortion services when the combination therapy mifepristone and misoprostol was included by WHO its Model List of Essential Medicines [5]. Mifepristone and misoprostol capsules can be self-administered to safely induce a discrete and non-invasive medical abortion in pregnant women up to 12 weeks of gestation.^1^ [6, 7] The combination was listed as a complementary medicine, following the opinion of the Expert Committee at the time that specialised health care facilities or services may be needed for its safe and effective use [8]. Moreover, WHO’s 2012 guidelines on safe abortion sent a strong message from an UN agency by endorsing the medical evidence and compelling human rights rationale for providing safe abortion [7]. Taken together, these endorsements from the public health community further legitimised medical abortion as an important clinical choice for women- one that should be part of a basic minimum package of medicines and available in functioning health systems.

In 2005, WHO’s then-Director General added a note to the entry on the Model List that read ‘[for use] where permitted under national law and where culturally acceptable’ (p. 37) [5, 9]. Such a conditionality risks offering a loophole to governments wary of embracing medical abortion, or abortion in general. Mifepristone and misoprostol’s addition to WHO’s Model List was lauded as an opportunity for medical abortion to reach women in rural and low-resourced settings who may otherwise be unable to seek safe surgical abortion [9]. In 2006, the more specific Interagency List of Essential Medicines for Reproductive Health also included mifepristone and misoprostol with the same adjacent note [10].

In 2016, the Committee on Economic, Social and Cultural Rights (CESCR), a collection of human rights experts tasked with interpreting these rights, asserted that abortion services are an integral part of the right to health in its ground-breaking interpretation of the right to sexual and reproductive health, General Comment No. 22 (para. 56-57) [11].

Since WHO’s initial move to improve access to mifepristone and misoprostol, mounting evidence of their safety combined with the evolution in human rights law now demand a revised approach. However, the status of mifepristone and misoprostol is unchanged in WHO’s 20th Model List of Essential Medicines published in March 2017 [12]. The time has come for WHO to remove the restriction of specialist supervision and to move the combination to the core list of the Model List of Essential Medicines and removing the qualifications.

These moves would send a strong signal to Member States - many of which are signatories to the International Covenant on Economic, Social and Cultural Rights (ICESCR) and therefore have the responsibility to protect and promote the right to health - to improve coherence between their international commitments and domestic fulfilment of the right. Enhancing policy coherence for sustainable development is a target in Sustainable Development Goal no. 17. These issues warrant thoughtful discussion by WHO’s Expert Committee on Selection and Use of Essential Medicines and support from the broader sexual and reproductive health and rights community.

In the following sections of this paper we first explore the integration of the public health and legal frameworks in light of the CESCR’s recent interpretation of sexual and reproductive health. Thereafter the arguments for two recommendations are presented: 1) mifepristone and misoprostol should be reconsidered as ‘core’ essential medicines rather than ‘complementary’ products; and 2) universal access to these medicines should be in line with human rights law and not unduly restricted by domestic law or cultural practices.

## Discussion

### Integrating the public health and legal frameworks for sexual & reproductive health

#### Essential medicines for medical abortion

Domestic governments can greatly improve the efficient use of health resources by selecting a narrow list of essential medicines, which are defined as ‘those that satisfy the priority healthcare needs of the population’ (p. 15) [13]. According to WHO, essential medicines are used for disease prevention, treatment, and control, and are applicable to most chronic and acute diseases. WHO’s definition states that essential medicines should be available in a well-functioning health system to all who need them, at a price the patient and the community can afford. When selecting which medicines are essential the WHO pays due regard to disease prevalence, but also to efficacy, safety and comparative cost-effectiveness [13].

WHO’s Model List of Essential Medicines is a standard-setting instrument for national medicines selection with a strong normative influence on decisions about medicines supply, distribution, and reimbursement. Described as an advocacy tool with an ‘operational, educational, and symbolic’ character, the Model List is simultaneously a guide for policy makers and program managers to a priority medicines requiring attention, an educational tool for decision makers and healthcare professionals about medicines selection and use, and a symbol of ‘worldwide recognition’ for key medicines [14, 15]. Although the Model List is not legally binding on governments, its authority directs policies ranging from medicines production to reimbursement decisions, even in a national court of law.

WHO’s guidelines on safe abortion adopt and promote a human rights-based approach to related laws and policies. The guidelines point to the growing medical evidence and compelling human rights rationale for providing safe abortion. WHO has recognised that almost every abortion-related death and disability could have been prevented through the provision of safe, legal induced abortion and care for complications (p. 1) [7]. In that line, WHO recommends that laws and policies on abortion should protect women’s health and their human rights and that regulatory, policy and programmatic barriers that hinder access to and timely provision of safe abortion care should be removed (p. 9) [7].

#### Human right to sexual & reproductive health

The right to health, affirmed most prominently in the 1966 International Covenant on Economic, Social, and Cultural Rights, requires governments to take action to assure maternal, child, and reproductive health. In 2000, human rights experts on the Committee on Economic, Social, and Cultural Rights (CESCR) adopted an authoritative interpretation of States’ responsibilities to assure to all the highest attainable standard of health, General Comment No. 14. In this document measures to combat maternal morbidity and mortality include ‘sexual and reproductive health services, including access to family planning, pre- and post-natal care, emergency obstetric services’ (para. 14) [16].

In 2016, the Committee on Economic, Social, and Cultural Rights extensively addressed States’ legal duties the right to sexual and reproductive health in its General Comment No. 22. This Comment affirms that this right is an integral part of the right to health- and is interdependent on a series of human rights to life, personal dignity, and others- that has enjoyed longstanding recognition based on already existing international human rights instruments. State parties to the ICESCR have the obligation to respect the right to sexual and reproductive health, which includes not limiting or denying access to health services such as abortion, or maintaining laws or practices that criminalise abortion, or requiring third-party authorisation to access contraception or abortion or excluding services such as abortion from publicly or donor- funded programmes. Therefore, States parties are duty-bound to facilitate access to safe abortion services including by aligning domestic law and healthcare packages with current human rights standards.

The Convention on the Elimination of All Forms of Discrimination Against Women (CEDAW) states that affirmative measures need to be taken to eliminate discrimination against women in the field of health care in order to ensure, on a basis of gender equality, access to health care services, including those related to family planning (p. 13) [17]. General Recommendation No. 24 forbids states from restricting access to health services or clinics to women “because they are women” or criminalise health services that only women need or punish women who seek those services [18]. The UN Committee on the Elimination of All Forms of Discrimination Against Women has repeatedly considered “that failure of a State party to provide services and the criminalisation of some services that only women require is a violation of women’s reproductive rights and constitutes discrimination against them.” [19].

Fulfilling this right bestows upon States the core obligation to provide medicines essential for sexual and reproductive health based on the WHO Model List of Essential Medicines (para. 49(g)) [11]. General Comment No. 22 specifically requires States to ensure the availability of essential medicines for abortion and post-abortion care (paras. 13 & 49(g)) [11]. The prominence of the Model List in General Comment 22 and other human rights jurisprudence illustrates its global authority and capacity for normative influence on Member States.

### Removing the ‘complementary’ designation: mifepristone and misoprostol qualify as ‘core’ essential medicines

Evidence substantiating mifepristone and misoprostol’s safety no longer warrants specialist medical care and justifies moving the combination therapy to the core list of essential medicines.

By 2005, mifepristone and misoprostol had been proven safe, effective and convenient to induce medical abortion. As the evidence base pointed to higher risks of minor adverse effects, such as longer duration of the bleeding compared to surgical abortion, WHO recommended the combination therapy be used under close medical supervision. Thus, mifepristone and misoprostol were entered on WHO’s complementary list. A complementary essential medicine is ‘for priority diseases which are efficacious, safe and cost-effective but not necessarily affordable, or for which specialised health care facilities or services may be needed’ (p. 20) [13].

The notion that these drugs warranted additional safety measures stems from the dearth of evidence from outside a research setting in the early days after its approval [20]. While the inclusion of these drugs in the complementary list was once likely a precautionary measure in a data-poor age, several studies have rendered such precaution unnecessary. Several studies confirmed that (i) abortion induced with misoprostol is safer than when induced by other means, and (ii) a reduction in the complications of unsafe abortion is observed over time, in parallel with an increase in the sales of misoprostol [21–24]. Moreover, the Mifeprex Risk Evaluation and Mitigation Strategy (REMS) Study Group affirms that a wealth of evidence now demonstrates mifeprostone’s effectiveness and safety [20]. Such substantive evidence exists that the Mifeprex REMS Study Group has recently called to revise the set of safety restrictions imposed on mifepristone and misoprostol in the US, stating that the safety measures are disproportionate to the low safety risks, and in some cases, the measures are not legitimate to achieve their objective [20].

In the 2012 WHO publication titled *Safe Abortion: Technical and Policy Guidance for Health Systems,* mifepristone and misoprostol are not only endorsed as a safe and reliable combination therapy for medical abortion, but also exempt from routine follow-up care in the absence of complications (p. 3) [7]. Aiken et al. recently reported low rates of adverse events experienced by women who received self-sourced medical abortion through telemedicine. In this study women self-identified potentially serious adverse events and most sought medical attention when advised to do so; no deaths were reported [25].

Reclassifying mifepristone and misoprostol as ‘core’ essential medicines on the Model List sends a clear message to WHO Member States that these therapies do not require specialist medical care for their safe and effective use. However, maintaining mifepristone and misoprostol on the complementary list in the absence of compelling safety concerns would indulge the trend to over-medicalise abortion services, which has historically obstructed access the service [26].

### Subjugating essential medicines for medical abortion to domestic law: an incoherent approach

Subjecting the use of mifepristone and misoprostol for medical abortion to national law is redundant, as presumably all governments exercise their discretion to use medicines on the Model List in a manner congruent with their domestic laws. More worrying is the fact that in 2005 the global agency for health policy, WHO, saw fit to specify that only medicines for medical abortion and emergency contraception in emergency health kits for refugee camps must comply with domestic law [9]. Curiously, this legal caveat was not explicitly stated in relation to other contentious therapies, such as methadone for drug addiction (p. 33-34) [5].

Removing the guidance that mifepristone and misoprostol use must comply with national law will limit States’ margin of discretion to sidestep their human rights obligations. The right to health standards clarified in the 2016 General Comment No. 22 indicate that national law must be brought in line with human rights obligations [27]. Governments have the obligation under human rights law to repeal or eliminate laws, policies and practices that criminalise, obstruct or undermine an individual’s or a particular group’s access to health facilities, services, goods and information, including abortion (para. 35) [11]. Moreover, scholars including those on the Lancet Commission on Women and Health emphasise the need for ‘an enabling social, legal, and regulatory environment’ to respond to women and girls’ health needs and rights [1]. The Commission on the Status of Women continues to demand that states strengthen their normative, legal, and policy frameworks in this field [28].

Domestic law does not universally embrace medical abortion [29–31]. Both human rights and public health standards should complement one another and encourage Member States to move towards a coherent approach.

Extensive literature shows that barring abortion does not stop women from terminating unwanted pregnancies, it only makes abortion considerably more dangerous (p. 32) [7]. Unsafe abortion mainly endangers women in countries where it is highly restricted by law and women in countries where, although legally permitted, safe abortion is not easily accessible [2]. Clandestine abortions contribute substantially to maternal morbidity and death worldwide [32]. Moreover, patients seeking safe abortion to end an unwanted pregnancy are often marginalised and can be made vulnerable by restrictive national abortion laws or inaccessible abortion services. It is precisely these patients who WHO’s Model List should strive to serve by unequivocally endorsing mifepristone and misoprostol as essential components of every health system.

Reducing the global maternal mortality ratio is one of the targets of the Sustainable Development Agenda adopted by the UN and mortality caused by unsafe abortion has long been one of the obstacles to its achievement. Even without concerted efforts to make these medicines more available, women worldwide are using them and they have saved thousands of lives [33–35]. The possibilities offered by mifepristone and misoprostol to prevent these avoidable deaths and health consequences should be accessible to all who can benefit from them.

Suggesting that restrictive domestic law on abortion justifies governments’ overt disregard or subtle apathy for universal access to mifepristone and misoprostol is incongruous with current human rights standards. Adopting domestic laws consistent with international treaties they have ratified demonstrates a government’s commitment to realise sexual and reproductive health and rights, while maintaining restrictions or expanding them constitute impermissible barriers and potentially a retrogressive measure that violates international human rights law [28]. Legal codification, a recognised indicator of these rights, may be the first step in improving the respect, protection and fulfilment of these rights in practice [36].

WHO has long recognised that the safety of abortion is directly associated with less restrictive legal settings. In its 2012 publication *Safe Abortion: Technical and Policy Guidance for Health Systems*, WHO comments on the public health and human rights rationale stating ‘[w]here legislation allows abortion under broad indications, the incidence of and complications from unsafe abortion are generally lower than where abortion is legally more restricted.’ (p. 17) [7]. The report continues to cite recommendations to States from UN committees to ‘reform laws that criminalise medical procedures that are needed only by women and that punish women who undergo these procedures, both of which are applicable in the case of abortion’ (p. 89 & box 4.1) [7]. Unequivocally WHO affirms ‘Given the clear link between access to safe abortion and women’s health, it is recommended that laws and policies should respect and protect women’s health and their human rights’ (p. 90) [7].

A coherent public health and human rights approach would confer an unfettered endorsement to essential medical abortion medicines, regardless of any possible limitations in domestic law and consonant with WHO’s own mandate to advance the right to health and its policy guidance supporting safe abortion.

###  Repeal the cultural acceptability limitation to universalise access to essential medicines for medical abortion

The WHO Model List qualifies the use of essential medicines for medical abortion where they are culturally acceptable. This limitation should also be removed for the following three reasons.

First, the CESCR identifies that health services must be culturally acceptable and respectful (para. 12(c)) [16]. It is reasonable to expect that States parties observe the CESCR’s guidance in the regular discharge of their responsibilities. Therefore, an explicit caveat in the WHO Model List about the use of any essential medicine in line with cultural norms is redundant.

Second, cultural acceptability of health services does not supersede States’ ‘core obligation’ to provide essential medicines. The CESCR’s interpretation affirms ‘core obligations’ as the nucleus of the right to health [16, 37]. Core obligations are fundamental basic minimums that must be realised to give meaning to the right to health; they serve as the foundation on which other aspects of the right are built [38, 39]. Additionally, the CESCR states that ideologically-based policies or practices, such as the refusal to provide services based on conscience, must not be a barrier to accessing services (para. 14) [11]. Therefore, a human rights approach suggests States are not justified in denying the provision of ‘core’ health services, such as essential medicines for medical abortion, when they do not conform with some restrictive cultural norms.

Third, numerous UN bodies have long acknowledged that violations of women’s right to health, such as lack of access to health goods and services, are often justified by references to culture or religion. Cultural acceptability is an element of the right to health that guides its shape in response to the local context but should not be misused to challenge the universality of women’s rights [16]. The UN Special Rapporteur in the field of cultural rights has recently warned of extremism that rejects the universality of human rights, aims to limit the enjoyment of women’s human rights and restrict sexual and reproductive rights under the label of ‘purity’ and ‘modesty’ [40]. States have an obligation to eradicate cultural practices that result in human rights violations such as barriers to access essential medicines for reproductive health [16, 17]. Therefore, the WHO Model List should not subordinate essential medicines for medical abortion to their cultural acceptability. Instead, those cultural practices should be modified in order to advance universal access to sexual and reproductive health.

## Conclusion

Ending the silent pandemic of unsafe abortion is an urgent public health and human rights imperative [2, 41]. WHO should align the pharmaceuticals entry for medical abortion on its Model List with the scientific evidence, human rights standards, and its own more recently developed policy guidance. The safety and efficacy of first trimester medical abortion with mifepristone and misoprostol is now better documented than when the combination was first introduced to the Model List [7]. Providing essential medicines for sexual and reproductive health is a core human rights obligation. In addition, access to such important life-saving drugs must not be explicitly nor implicitly subjected to restrictive domestic laws or cultural norms. Any restriction to access mifepristone and misoprostol for medical abortion before 12 weeks of gestation would be tantamount to a violation of international human rights law. WHO’s leadership is imperative to signal that mifepristone and misoprostol are part of a legitimate and inviolable reproductive healthcare package that must be strengthened, rather than subjugated, by national law or cultural practices. WHO’s Expert Committee on Selection and Use of Essential Medicines is in a unique position to bridge the gap between human rights law and WHO policy to respect and fulfil sexual and reproductive health in practice.

## Abbreviations

CEDAW: Convention on the Elimination of All Forms of Discrimination Against Women
CESCR: Committee on Economic, Social and Cultural Rights
ICESCR: International Covenant on Economic, Social and Cultural Rights
REMS: Risk Evaluation and Mitigation Strategy
WHO: World Health Organization

## Footnotes

1 Misoprostol can be administered vaginally or sublingually. According to WHO Guidelines on Safe Abortion, strong evidence supports the use of the combination therapy up to 9 weeks of gestation and after 12 weeks of gestation [7]. Evidence from Uruguay indicates the combination therapy can be safely used up to 12 weeks of gestation [6].
